# Quality of care in type 2 diabetes in Iran; a cross-sectional study using patient-level data

**DOI:** 10.1186/s12902-022-01034-2

**Published:** 2022-05-17

**Authors:** Majid Davari, Yahya Bayazidi, Abbas Kebriaeezadeh, Alireza Esteghamati, Fatemeh Bandarian, Zahra Kashi, Adele Bahar, Sepideh Yousefi

**Affiliations:** 1 Department of Pharmacoeconomics and Pharmaceutical Administration, Faculty of Pharmacy, Tehran University of Medical Sciences, Tehran, Iran; 2 Endocrinology and Metabolism Research Center, Vali-Asr Hospital, School of Medicine, Tehran University of Medical Sciences, Tehran, Iran; 3 Endocrinology and Metabolism Research Center, Tehran University of Medical Sciences, Tehran, Iran; 4 Diabetes Research Center, Mazandaran University of Medical Sciences, Mazandaran, Iran; 5 Pharm-D, Faculty of Pharmacy and Pharmaceutical Science, Islamic Azad University of Medical Sciences, Tehran, Iran

**Keywords:** Quality of Care, Type 2 Diabetes, Optimal Control, ABC Care, HbA1c, Iran

## Abstract

**Background:**

Appropriate service delivery, access to high quality of cares and optimal management of type 2 diabetes mellitus (T2DM) can decrease the risk of micro and macro vascular complications and mortality. Therefore, monitoring the quality of diabetes care, including keeping glycemic levels at an optimal level, is crucial. The aim of this study was to evaluate processes and outcome-related quality of care indicators, in T2DM using retrospective patient-level data from 2013 to 2017 in 15 Tertiary Diabetes Care Centers in Iran.

**Method:**

A retrospective observational study was conducted among 1985 T2DM patients at public, semipublic and private diabetes centers. Annual tests for HbA1c, serum lipid (LDL), and screening for nephropathy were used to evaluate process-related indicators; and intermediate biomedical markers including HbA1c, blood pressure (BP), and LDL cholesterol, were used to assess outcome-related indicators.

**Results:**

Data were extracted from 15 diabetes centers in five provinces in Iran. 62.7% of the patients were female, and the mean duration of diabetes in the patients was 14.7 years. Evaluation of process-related indicators showed that only 9% of patients took the HbA1c test. The percentage of the patients without annual low-density lipoprotein (LDL) test decreased from 13% in 2013 to 7% in 2017. The results of achieving to all indicators concurrently (ABC care) showed that less than 2% of the patients met the criteria of optimal process-related quality indicators.

The mean percentage of the patients with HbA1c under 7%, blood pressure (BP) less than 130/80 mmHg, and LDL less than 100 mg^/dl^ in the selected provinces were 32.4, 55, and 71 respectively. However, the average of total achievement in ABC goals was 14.2%.

**Conclusion:**

Our findings showed that the management of T2DM in all selected provinces was far from the optimal control in both processes and outcome-related indicators and therefore needs serious consideration and improvement.

## Background

Studies show that the Middle East will experience a massive increase in diabetes burden among the rest of the world in the coming years. Most of this increase will occur in people aged 45 to 64 who are economically active population of the community. In Western countries, however, most people with diabetes are over 65 years old who are economically less active or inactive [1].

The prevalence of type 2 diabetes mellitus (T2DM) in Iran has continuously increased in past years due to population growth, aging, urbanization, obesity, and sedentary life style [2, 3]. It is estimated that 11.4% of Iranian have diabetes [4], although about 40 percent of them have not been diagnosed yet [5]. This continuous and considerable increase in the prevalence of T2DM can be a sign of the high health and economic burden of disease in Iran, specifically when considering the impact of diabetes-related complications [6, 7]. Esteghamati has shown a relative improvement in health outcomes in T2DM patients in Iran in the past ten years [8]. However, a few studies have shown the opposite results as well [9, 10].

The delivery and access to a high quality of care and regular physician consultations can decrease the risk of micro and macro vascular complications and mortality [11]. Therefore, monitoring the quality of diabetes care including indicators of process and outcome is crucial [12–15]. American Diabetes Association (ADA) currently recommends that HbA1c measurements be applied at least semiannually in patients with adequate glycemic control; and quarterly for those who were not meeting glycemic targets and for whom the therapeutic regimen has changed. Clinical guidelines also suggest that for patients who have not had a HbA1c test for the past year or even three months, the physician should promptly set blood glucose targets and request a HbA1c test for subsequent visit [16]. If the situation is still uncontrolled, both regular HbA1c testing and medication evaluation should be considered [17].

Indicators for measuring the quality of care include two main types; process and outcome-related indicators [18, 19]. Process-related indicators focus on health care utilization, such as the number of annual HbA1c tests. However, outcome-related indicators focus on achieving an appropriate level of intermediate biomedical markers, for instance, the percentage of patients achieving the desired level of HbA1c.

The aim of this study was to evaluate the quality of care through assessing both process and outcome related indicators, in patients with T2DM using patient-level data in Tertiary Care diabetes centers during the five years (2013–2017) in Iran.

## Methods

### Study design

Clinical data extracted from the patients’ profiles in 15 main diabetes centers including public, semi-public (centers related to the social security insurance organization), and private diabetes centers in Iran. We performed a retrospective data analysis using patient-level data in a cross-sectional study. We assessed the process and outcome-related indicators of the patients over a 5-year period (from 2013 to 2017).

### Sampling method

Two steps sampling selection was applied to recruit the needed sample size. In the first step, cluster sampling method was applied to select 5 provinces (clusters); Tehran, Isfahan, Yazd, Mazandaran, and Kurdistan. Tehran and Isfahan provinces were two metropolises (23% of Iran's total population lived in these two provinces in 2016). These provinces have better access to specialized health care services compared to others. Yazd had the highest prevalence of diabetes (16.3%) in the whole country. The family physician program is running in Mazandaran, and Kurdistan was one of the deprived provinces regarding access to health care.

In the second step, 15 main diabetes care centers selected from the provinces. The subjects were selected from each center using random sampling method based on the patient identification number. In centers that had a statistically small population, the total population was included. Inclusion criteria included diagnosis of type 2 diabetes, use of anti-diabetic drugs, and having an active file in the cross-sectional schedule.

### Research ethics approval: human participants

Ethics approval was obtained from the Ethics Committee (IR.TUMS.PSRC.REC.1396.1991) at Tehran University of Medical Sciences.

### Statistical analysis

Statistical analysis was performed using the STATA software version 14 for Windows (Stata Corp., College Station, TX, US).

Based on the results of normality tests, continuous variables (i.e. blood pressure, duration of disease, glycemic, and lipid indices) are presented either as mean ± standard deviation (SD)/standard error. Categorical outcomes (i.e. meeting the preset glycemic, blood pressure and lipid control targets) are demonstrated as proportions (95% confidence interval [95% CI]) or mean ± S.E.M. For comparing multivariate sample means, we used Multivariate Analysis of Variance (MANOVA) analysis.

### Process-related quality indicators

To find out time-varying process indicators, we extracted the following aspects (19, 20):

- For blood glucose control, a quarterly glycated hemoglobin (HbA1c) test was considered as a standard limit based on clinical guidelines.
- For lipid control, serum lipid (low-density lipoprotein (LDL)) testing at least once a year was considered appropriate control.
- For nephropathy screening, one or both of the following tests were considered appropriate: a urine protein test or a urine albumin test.

### Outcome-related quality indicators

To measure the achievement of outcome indicators, we used HbA1c, Blood Pressure and Cholesterol (ABC) Care [19], in which ADA has put a massive emphasis on the control and achievement of triple goals concurrently.

- For blood glucose control, HbA1c < 7% was considered as the standard limit.
- Blood pressure < 130/80 mm Hg was considered a standard limit to control blood pressure profiles.
- To control the lipid profile, LDL < 100 mg/dL considered as the standard limit

Finally, the percentage of patients with simultaneous achievement of ABC care goals was evaluated [17].

### Patient and public involvement

All of the patients provided written informed consent.

## Results

The results and analysis are based on data from 1984 patients with T2DM extracted from public, semi-public, and private diabetes centers in five provincial capital cities (Tehran, Isfahan, Yazd, Mazandaran, and Kurdistan).

### Demographic indicators

More than half of the patients (62.7%) were female, and the mean duration of diabetes in the patients was 14.7 years (± 6.62 years). Overweight and obesity were common among the patients and approximately 83% of them had an inadequate body mass index. 67.6% of the patients were in the age group of 25 to 65 years (Table 1).

**Table 1:** Demographic information of T2DM patients

		**Tehran**	**Isfahan**	**Yazd**	**Kurdistan**	**Mazandaran**	**Total**
Female (sex percent)		64	62	60	60	61	62.7
Number of patients		395	400	395	400	395	1985
mean diabetic age (years)		15.84	14.34	14.46	15.28	13.71	14.72
Average age (years)		62.85	63.45	60.55	61.7	63.11	62.33
Body Mass Index	kgm^2^	0.0	0.1	0.1	0.0	0.1	0.06
	18.5 – 24.9 kg/m^2^	18	16	17	15	20	17.2
	25.0–29.9 kg/m^2^	45	42	40	50	46	44.6
	≥30 kg/m^2^	37	41.9	42.9	35	33.9	38.14
Age	<45	5	10	7	6	9	7.4
	45-65	65	55	60	62	59	60.2
	>65	30	35	33	32	32	32.4

### Process-related quality indicators

For blood glucose control, we evaluated the percentage of the patients with an HbA1c test greater than/equal to 1 per three months and the percentage of the patients without an HbA1c test per year. The results showed that only 8% and 9% of the patients in 2013 and 2017 had taken quarterly HbA1C test. However, the percentage of the patients without an HbA1c test per year decreased from 13% in 2013 to 2% in 2017. The mean number of annual HbA1c tests was slightly increased from 1.6 in 2013 to 1.9 in 2017 (Table 2).

**Table2:** Process-related quality indicators

Index	2013	2014	2015	2016	2017
**Glycemic control monitoring**					
Percentage of HbA1c test ≥ 1 per three months (Number)	8 (158)	9 (178)	10 (198)	10 (198)	9 (178)
Percentage of patients without HbA1c test annually (Number)	13 (257)	10 (198)	7 (138)	6 (119)	2 (40)
Mean HbA1c test per year per patient	1.62	1.71	1.82	1.86	1.89
**Lipid profile monitoring**					
Mean Serum lipid test ≥ 1 per year	1.50	1.65	1.63	1.68	1.59
Percentage of patients without LDL test (number)	13 (258)	8 (159)	8 (159)	7 (139)	7 (139)
**Nephropathy screening**					
Percentage of urine protein test ≥ 1 per year (number)	46(913)	45(893)	43(854)	47(933)	42(834)
Mean Urine protein test per year per patient	0.55	0.54	0.55	0.61	0.53
**ABC care**					
Percentage of ABC care^1^ (Number)	0.008 [15]	0.015 [20]	0.017 [21]	0.018 [22]	0.017 [23]
Percentage of ABC care^2^ (Number)	0.28 (547)	0.28 (549)	0.30 (588)	0.33 (652)	0.29 (565)

1. HbA1c test ≥ 1 per three months and lipid test ≥ 1 per year and urine protein test ≥ 1 per year, 2. HbA1c test ≥ 1 per six months and lipid test ≥ 1 per year and urine protein test ≥ 1 per year

Table 2 shows that the percentage of the patients without annual LDL test decreased from 13% in 2013 to 7% in 2017. However, the mean number of LDL test per person per year did not changed significantly during the study period.

About 45 percent of the patients had taken one of two tests for urine protein or urine albumin, and the mean number of urine protein tests was 0.5 test per year.

The results of the evaluation of all indicators together (HbA1c test ≥ 1 per three months and lipid test ≥ 1 per year and urine protein test ≥ 1 per year (ABC care)) showed that less than 2% of the patients met the criteria of optimal process-related quality indicators. However, if we consider a moderate ABC care (HbA1c test ≥ 1 per six months and lipid test ≥ 1 per year and urine protein test ≥ 1 per year), the simultaneous achievement will increase to 29% (Table 2).

### Outcome-related quality indicators

The average percentage of the patients with HbA1c level under 7% in selected provinces showed that this percentage varied from 22 to 43%. Yazd had a lowest rate (22%) and Kurdistan had a highest rate (43%) of HbA1c control during the study time. The trends in the provinces showed that this percentage in Yazd increased from 18% in 2013 to 24% in 2017; but in Kurdistan it decreased from 46% in 2013 to 40% in 2017. However, the average of total HbA1c control was 32.4% during the study time (Table 3).

**Table 3:** Percentage of patients with HBA1c level < 7% during 2013–2017

Year	Tehran	Isfahan	Yazd	Kurdistan	Mazandaran	Mean
2013	31	31	18	46	32	32.4
2014	32	32	18	48	37	34.1
2015	32	34	20	45	33	33.7
2016	27	35	28	38	25	30.6
2017	29	33	24	40	27	31.2
Total	30	33	22	43	31	32.4
*P*-Value for Trend	0.068	0.064	0.038	0.021	0.001	0.061

The average percentages of the diabetes patients with blood pressure (BP) target (< 130/80 mmHg) in the selected provinces ranged from 36 to 75%. The best BP control was in Isfahan province (75%) and the worst BP control was in Yazd province (36%). The P-value of trends in the provinces showed that only Mazandaran has changed significantly during the 5 years of the study. The average of total BP control was 55% (Table 4).

**Table 4:** Percentage of patients with BP level < 130/80 mmHg during 2013–2017

Year	Tehran	Isfahan	Yazd	Kurdistan	Mazandaran	Mean
2013	55	76	30	60	58	56
2014	60	75	38	60	53	57
2015	53	76	41	59	56	57
2016	48	76	37	64	45	54
2017	41	73	35	55	42	49
Total	51	75	36	60	51	55
*P*-Value for Trend	0.017	0.062	0.037	0.036	0.001	0.037

The percentage of the patients who achieved the LDL target (< 100 mg^/dl^) was fluctuated from 63 to 76%. Mazandaran province had the highest rate of LDL control (76%) and Yazd province had the lowest rate (63%). However, the P-value of the trends in the provinces showed that none of them changed significantly during the 5 years of the study. The average of total LDL control was 71% (Table 5).

**Table 5:** Percentage of patients with LDL level < 100^ mg/dl^ during 2013–2017

Year	Tehran	Isfahan	Yazd	Kurdistan	Mazandaran	Mean
2013	70	72	46	61	69	64
2014	76	76	61	57	72	68
2015	72	66	70	70	78	71
2016	76	72	73	74	78	74
2017	81	78	68	77	86	78
Mean	75	72	63	67	76	71
*P*-Value for Trend	0.041	0.047	0.037	0.036	0.021	0.036

### Achieving ABC targets

Our findings show that the simultaneous achievement of ABC goals for diabetics, according to the ADA guideline, varied significantly between the provinces. The lowest achievement was related to Yazd (5% on a 5-year average), and the highest achievement belonged to Kurdistan (20% on a 5-year average). Nonetheless, the P-value of the trends in the provinces show that none of them had a significant change in achieving the ABC goals during the study period. The average of total achievement in ABC goals was 14.2% (Table 6).

**Table 6:** Percentage of patients with simultaneous achievement of ABC goals^1^ during 2013–2017

Year	Tehran	Isfahan	Yazd	Kurdistan	Mazandaran	Mean
2013	16	19	3	18	12	13.6
2014	18	20	5	19	12	14.8
2015	14	19	5	19	16	14.6
2016	12	21	7	20	10	14.0
2017	10	21	6	20	12	13.8
Mean	12	19	5	20	14	14.2
*P*-Value for Trend	0.078	0.066	0.061	0.063	0.052	0.06

1. ABC goals: HBA1c level < 7% and BP level < 130/80^ mmHg^ and LDL^mg/dl^ level < 100

## Discussion

The aim of this study was to evaluate the quality of care (process and outcome) for T2DM patients based on both process and outcome indicators. We conducted institution based cross-sectional study using patient-level data in public, semipublic and private diabetes centers during the five years (2013–2017) in Iran.

The distribution of the selected provinces was wide enough to ensure that we had different types of the patients in our samples.

Our findings show that most patients (67.6%) were under 65 years old. This is almost twice as many as in European countries [1]. This could confirm that diabetics in Iran, and perhaps in many other developing countries, are younger than diabetics in European countries. This could indicate the importance of assessing the quality of care for diabetics and its potential impact on reducing the burden of diabetes in developing countries.

### Process-related quality indicators

Our findings show that less than 10% of the patients received appropriate number of HbA1c services and did not change statistically significantly during the study period. Previous studies showed that only 6.4% of the patients had more than or equal to one HBA1c test per year [11]. Given the 9-year interval between 2 studies, a 2.6% increase in the number of patients with appropriate testing is not an acceptable progression. Nonetheless, decreasing the percentage of the patients without an HbA1c test from 13% in 2013 to 2% in 2017 can be considered as improving the quality of care for diabetics in Iran, but it is still far from the guideline’s recommendations.

The conditions for measuring the level of LDL in the patients were significantly better. Our findings showed that 93% of the patients met the minimum standards of cares for measuring the level of LDL in diabetics’ patients.

About 57% of the patients did not meet the minimum standards for two urine protein or albumin tests. This percentage did not change significantly during the study time and within the provinces.

Our findings show that among the process-related indicators, LDL monitoring had the best situation within the indicators. Nonetheless, considering that the prevalence of T2DM in Iran is more than 11% and the number of diagnosed patients is estimated at more than 5.5 million, this 7% is still significant and should reduce to zero [4].

When all the indicators are considered together (ABC care), the inadequacy of care will be more obvious. The results of ABC care showed that less than 2% of the patients met the criteria of optimum care. This means that more than 98% of the patients did not receive the expected monitoring cares; which is very far from the guidelines’ recommendations [16]. However, if moderate ABC care is considered, the simultaneous achievement increases to 29%, which indicates that most patients have not yet received the recommended care (Table 2).

### Outcome-related quality indicators

The percentage of the patients with HbA1c level under 7% in selected provinces showed that although there were significant variations between the provinces (22 to 43%.), but in all of them, a small number of patients had the recommended level of HbA1c. The lowest percentage of good control occurred in Yazd province (Table 3), which has the highest prevalence of T2DM in Iran [24]. Although this percentage increased from 18 to 24% in 5 years, it is still very low. The P-Value for trends also confirms that the changes, except for Mazandaran province, were not statistically significant. The analysis of the results of Mazandaran province illustrates that the percentage of patients with good control of HbA1c decreased from 32 to 27% (Table 3). Therefore, the control status of HbA1c in Mazandaran province deteriorated significantly during the study. Since Mazandaran is one of the provinces in Iran where the family physician program is implemented, these results confirm that the family the physician program has not had a good impact on the management of T2DM.

The results of Tehran and Isfahan, two metropolises of Iran, illustrated that there was no statistically significant change (decrease/increase) in achieving optimal HbA1c levels between these provinces with other provinces.

A recent study showed that only 13.2% of diabetes in Tehran achieved the blood glucose control [8]. However, since the patient population included all types of diabetes, the results may not be completely comparable. Nonetheless, as more than 85% of these patients had T2DM, comparing the results of these two studies is somewhat acceptable.

Mohammed et al. showed that the proportion of American patients who achieved the recommended goals for diabetes care increased by 7.9% over 12 years. Nonetheless, 48.7% of the patients did not reach the target for glycemic control at the end of study [25]. Another study in Catalonia (2007- 2013) showed that the percentage of the T2DM patients with HbA1c less than 7%, increased from 52.2% to 55.6% in the study time [26]. Comparing these achievements with our results show that quality of care for patients with T2DM in selected provinces in Iran, with 67.6% of the patients out of control, needs more attention and development.

Examination of the pattern of antidiabetic prescription during the study time showed that no significant change were observed in the drug prescriptions. The details of the analysis of prescription pattern are discussed elsewhere [27]. This analysis showed that only 25% of the total antidiabetic medicines were insulins [27]. However, the ratio of insulin consumption in some European countries has varied from 30 to 50 percent [28].

Many studies have shown that insulins are more effective in managing T2DM than oral medications, particularly in patients with HbA1c above 7% [29, 30, 31, 32]. The lack of change in the pattern of prescribing antidiabetic drugs, despite the low rate of patients with the desired HbA1c level, indicates that physicians have not responded properly to the patients' HbA1c level.

Since the pattern of T2DM administration was almost the same between the provinces [27], the better results of diabetic patients in Kurdistan (43% of patients with good HbA1c level) can be attributed to their lifestyle and perhaps their genetics; but more study and information is needed to confirm this.

BP control of the diabetics (less than 130/80 mmHg) in the selected provinces also varied significantly (from 36 to 75%). The best BP control was in Isfahan province (75%) and the worst was in Yazd province (36%). However, the P-value of the trends in the provinces showed that, except for Mazandaran province, the trends of BP control did not change significantly during the 5-year study. The results of Mazandaran province illustrates that the percentage of patients with BP control decreased from 58 to 42% (Table 3). Therefore, the significance of BP trend in Mazandaran province has been in a negative direction. These results confirm that physicians in none of the provinces responded correctly to the patients’ BP levels.

Although the status of blood LDL levels was better than the previous two factors, the mean percentage of the patients who achieved the LDL control target (< 100 mg^/dl^) ranged from 63 to 76%. Mazandaran province had the highest rate of LDL control (76%) and Yazd province had the lowest rate (63%). However, the P-value of trends in the provinces showed that none of them had changed significantly in the study time. The average of total LDL control was 71% (Table 5) which is still far from the desired value. These findings also showed that physicians did not have appropriate prescriptions for the patient’s condition.

### Achieving ABC targets

Our findings show that the simultaneous achievement of ABC goals for diabetics, according to the ADA guideline, varied significantly between the provinces. The lowest achievement was related to Yazd (average 5% in 5 years), and the highest achievement belonged to Kurdistan (average 20% in 5 years). Nonetheless, the P-value for trends in the provinces show that none of the provinces had a significant change in achieving the ABC goals during the study period. The average of total achievement of ABC goals was 14.2% (Table 6).

Our results showed that in the last years of the study only 13.8% of the patients achieved the ABC targets. Although it shows a slight increase compared to the beginning of the study (13.6%), but it is not statistically significant (*p*-value > 0.05). The results of the study of Casagrande showed a significant improvement in diabetes care between 1988 and 2010 in the USA. It is showed that the percentages of people with ABC goals were increased from 1.7% in 1994 to 18.8% in 2010; and the increases were statistically significant [33].

Many studies have emphasized that diabetes is a complex chronic illness requiring multiple strategies beyond glycemic control including patients’ adherence to treatment and their appropriate lifestyle. Nonetheless, it is stated that the gap between the facts and the optimal condition of the patients are often related to physician practice""Barriers to effective management of type 2 diabetes in primary care: qualitative systematic review"" [34]. Our findings also confirm that the poor results of the management of T2DM in the selected provinces can be the inattentiveness of the physicians. Inadequate prescribing of required tests (Table 2) and failure to change the prescription according to the patients' needs [27] can confirm this statement.

## Conclusion

Our findings show that most T2DM patients are younger than diabetics in European countries and then potentially the burden of diabetes in Iran can be higher than European and Western countries, and this finding emphasized to priority of good clinical practice for diabetic’s population in Iran. The evaluation of process-related quality indicators illustrated that more than 90% of the patients received inappropriate number of HbA1c tests during the study period. Although these criteria were higher for LDL and urine tests, the ABC care goals showed that more than 98% of the patients did not receive quality process-related care.

The outcome-related quality indicators illustrated that around 70% of the patients had the optimal level of HbA1c. Although this percentage was higher for LDL and BP control level, the average of total achievement of ABC goals was 14.2%.

Our findings showed that the management of T2DM in all selected provinces was far from the optimal control in both processes and outcome-related indicators and therefore needs serious consideration and improvement. The outcomes were slightly better in Kurdistan province, a deprived province, probably because of their lifestyle and genetics.

### The design and methods of current study had several strengths

The data used in this study include laboratory measurements for diabetics over a 5-year period. We used a longitudinal design that can show the variable nature of laboratory measurements in diabetic patients over time. This gives us a better understanding of the quality of diabetes care compared to a simple cross-sectional design, which uses baseline laboratory values in their estimations.

### The limitations and possible source of biases

The study includes common warnings associated with retrospective studies. The electronic and documentary medical records did not include information on disease severity, lifestyle modifications, and physical activity.

## Data Availability

The datasets used and/or analyzed during the current study are available from the corresponding author on reasonable request. All methods were carried out in accordance with relevant guidelines and regulations.

## Abbreviations

T2DM: Type 2 diabetes mellitus
HbA1c(Hemoglobin A1c): Glycated hemoglobin
LDL: Low-density lipoproteins
BP: Blood Pressure
BMI: Body Mass Index
ABC: HbA1c, Blood Pressure and Cholesterol
